# A paclitaxel-hyaluronan conjugate (ONCOFID-P-B™) in patients with BCG-unresponsive carcinoma in situ of the bladder: a dynamic assessment of the tumor microenvironment

**DOI:** 10.1186/s13046-024-03028-5

**Published:** 2024-04-10

**Authors:** Anna Tosi, Beatrice Parisatto, Enrico Gaffo, Stefania Bortoluzzi, Antonio Rosato

**Affiliations:** 1 Immunology and Molecular Oncology Diagnostics, Veneto Institute of Oncology IOV-IRCCS, Via Gattamelata 64, 35128 Padova, Italy; 2 Department of Surgery, Oncology and Gastroenterology, University of Padova, Via Gattamelata 64, 35128 Padova, Italy; 3 Department of Molecular Medicine, University of Padova, Padova, Italy

**Keywords:** Bladder cancer, Macrophages, CD44, CD44v6, Predictive biomarkers, Hyaluronic acid

## Abstract

**Background:**

The intravesical instillation of the paclitaxel-hyaluronan conjugate ONCOFID-P-B™ in patients with bacillus Calmette-Guérin (BCG)-unresponsive bladder carcinoma in situ (CIS; NCT04798703 phase I study), induced 75 and 40% of complete response (CR) after 12 weeks of intensive phase and 12 months of maintenance phase, respectively. The aim of this study was to provide a detailed description of the tumor microenvironment (TME) of ONCOFID-P-B™-treated BCG-unresponsive bladder CIS patients enrolled in the NCT04798703 phase I study, in order to identify predictive biomarkers of response.

**Methods:**

The composition and spatial interactions of tumor-infiltrating immune cells and the expression of the most relevant hyaluronic acid (HA) receptors on cancer cells, were analyzed in biopsies from the 20 patients enrolled in the NCT04798703 phase I study collected before starting ONCOFID-P-B™ therapy (baseline), and after the intensive and the maintenance phases. Clinical data were correlated with cell densities, cell distribution and cell interactions. Associations between immune populations or HA receptors expression and outcome were analyzed using univariate Cox regression and log-rank analysis.

**Results:**

In baseline biopsies, patients achieving CR after the intensive phase had a lower density of intra-tumoral CD8+ cytotoxic T lymphocytes (CTL), but also fewer interactions between CTL and macrophages or T-regulatory cells, as compared to non-responders (NR). NR expressed higher levels of the HA receptors CD44v6, ICAM-1 and RHAMM. The intra-tumoral macrophage density was positively correlated with the expression of the pro-metastatic and aggressive variant CD44v6, and the combined score of intra-tumoral macrophage density and CD44v6 expression had an AUC of 0.85 (95% CI 0.68–1.00) for patient response prediction.

**Conclusions:**

The clinical response to ONCOFID-P-B™ in bladder CIS likely relies on several components of the TME, and the combined evaluation of intra-tumoral macrophages density and CD44v6 expression is a potentially new predictive biomarker for patient response. Overall, our data allow to advance a potential rationale for combinatorial treatments targeting the immune infiltrate such as immune checkpoint inhibitors, to make bladder CIS more responsive to ONCOFID-P-B™ treatment.

**Supplementary Information:**

The online version contains supplementary material available at 10.1186/s13046-024-03028-5.

## Background

The standard therapy for bladder carcinoma in situ (CIS) is represented by intravesical instillation of Bacillus Calmette-Guerin (BCG) that, however, can lead to intolerance or unresponsiveness [1].

Paclitaxel (PTX) is an antimitotic agent active against many cancers, including bladder cancer, but with several drawbacks [2]. To overcome PTX clinical limitations, a strategy relates to the conjugation of PTX to hyaluronic acid (HA) as a carrier, which offers several advantages in terms of biocompatibility, tolerability and solubility [3]. Indeed, HA-drug conjugates can efficiently bind to cancer cells overexpressing HA receptors, and exert strong antiproliferative and cytotoxic activity [4]. Among HA receptors, the most studied is CD44, a transmembrane glycoprotein overexpressed in several tumors. CD44 is encoded by 19 exons, with 9 of them undergoing alternative splicing and generating CD44 variants (CD44v), each of them activating different signalling pathways that in turn lead to distinct functions [5]. A PTX-HA formulation, namely ONCOFID-P-B™, has been reported to significantly increase CD44-dependent cellular uptake of the chemotherapy moiety in bladder cancer cell lines [4]. Moreover, ONCOFID-P-B™ has been already tested in BCG-refractory patients with bladder CIS [6], demonstrating high tolerability and achieving 60% of complete response (CR) following 6 weekly intravesical instillations. These positive observations were confirmed in the NCT04798703 phase I study [7], where the intravesical administration of ONCOFID-P-B™ for 12 consecutive weeks (intensive phase, IP) led to 75% of CR. Patients with a CR at this time point underwent a subsequent maintenance phase (MP) of 12 monthly instillations and at month 15 the CR rate was still 40%, thus supporting further clinical development for ONCOFID-P-B™. However, potentially predictive biomarkers of treatment response to ONCOFID-P-B™ require to be identified.

In the present study, we provide a detailed description of the tumor microenvironment (TME) of ONCOFID-P-B™-treated BCG-unresponsive bladder CIS patients enrolled in the NCT04798703 study. In particular, we focused on the composition and spatial interactions of tumor-infiltrating immune cells, and evaluated the expression of the most relevant HA receptors on bladder cancer cells. Moreover, the study provided a unique opportunity to monitor the therapy-induced changes in immune cell composition and HA receptors expression, ultimately leading to the identification of biological markers predictive of response to ONCOFID-P-B™ treatment.

## Methods

### Patient samples

**Fig. 1:**
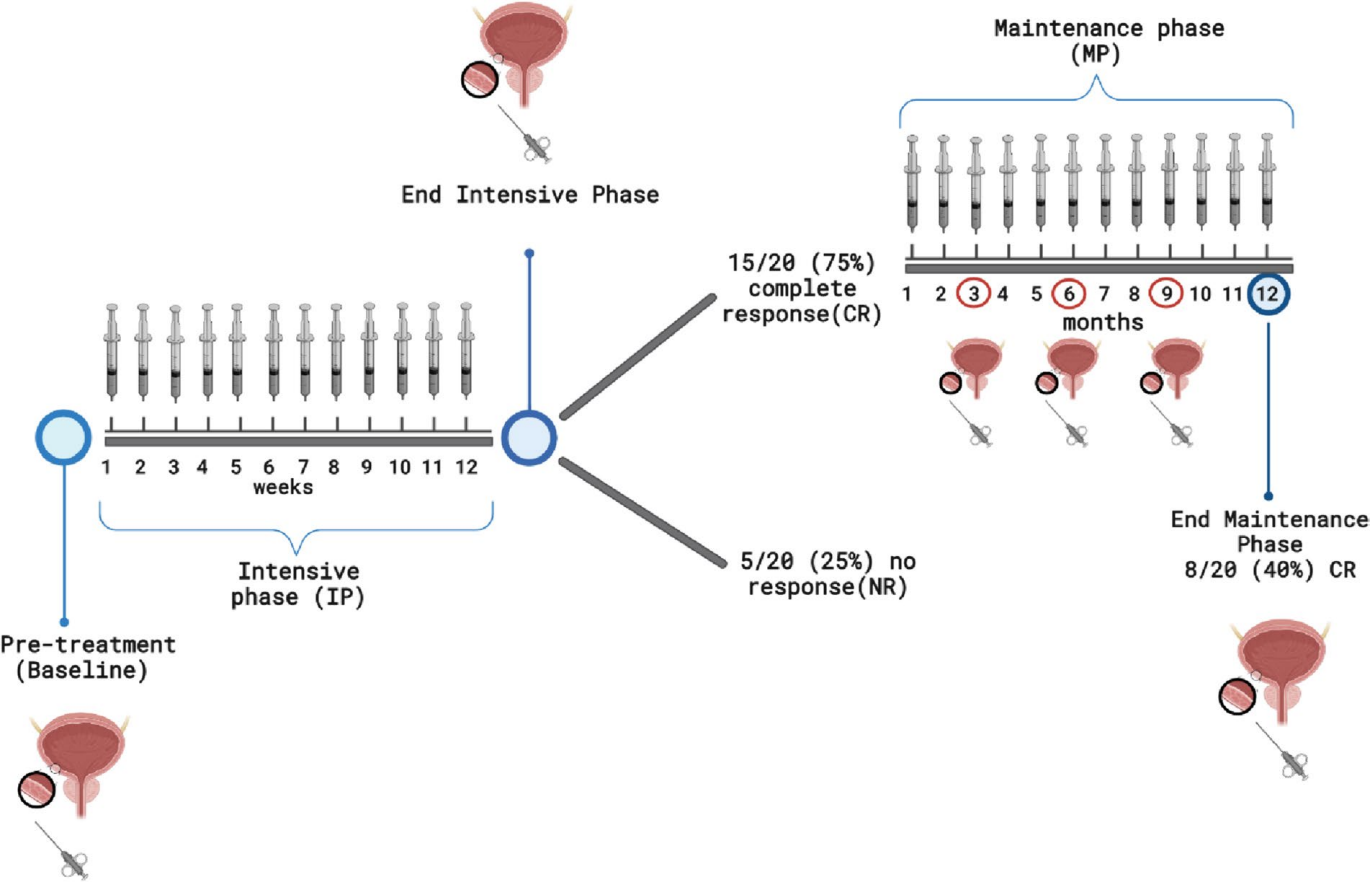
Trial profile and samples collection timeline. Created with BioRender.com

Based on the clinical study protocol [7], bioptic samples from urothelial mucosa were collected during the cystoscopy from 20 subjects with BCG-unresponsive CIS +/−Ta-T1, before ONCOFID-P-B™ (baseline) and after the 12-week IP. Patients who achieved a CR (defined as a negative cystoscopy including negative biopsy of the urothelium and negative cytology) after the IP entered a subsequent 12 monthly instillations MP. In such patients, additional biopsies were collected every 3-months to assess the duration of response (Fig. 1). We referred as non-responders (NR) whenever drug discontinuation occurred or a positive cystoscopy or cytology confirmed any evidence of persistent CIS, progression disease or relapse. Disease-free survival (DFS) was calculated from the beginning of the treatment to relapse or to the end of the treatment protocol, whichever first. After the IP, 15/20 patients achieved a CR and, among these, 8 patients still had a CR after the MP. The response rate of the study is 40%, and the median DFS is 12 months (95% CI 2.5-21.4). The clinical characterization of these patients has been previously reported [7]. Specimens were fixed in 10% buffered formalin solution for 24 hours and paraffin embedded. Only biopsies containing tumor tissue confirmed by a pathologist were considered and analyzed. The study was conducted according with Good Clinical Practice Guidelines, the World Medical Association Declaration of Helsinki, and the directives of the Committee of the Ministers of EU member states on the use of samples of human origin for research. All patients provided written informed consent. The trial protocol and all amendments were approved by the competent ethical committee at each participating institution [7]. Four normal bladder samples deriving from the Body Donation Program of the Institute of Human Anatomy of the University of Padova [8] were collected from anonymous donors who died from causes not attributable to bladder cancer, and matched gender and age characteristics of ONCOFID-P-B™-treated patients, and prepared as done for tumor biopsies (Supplementary_Table_1).

### Multiplex immunofluorescence

The immune TME and the expression of the most relevant HA receptors on cancer cells were analyzed by multiplex immunofluorescence (mIF) on sequential 4 μm-thick formalin-fixed paraffin-embedded (FFPE) tumor tissue sections using the Opal Polaris 7-Color Automated IHC Detection Kit (Akoya Biosciences, Marlborough, MA, USA). Two custom 9-color staining panels and one 4-color panel were carefully designed to characterize the subsets of tumor-infiltrating immune cells, and the expression pattern of HA receptors on cancer cells. For each marker of the panels, the staining condition were optimized using monoplex stained slides from positive control tissues, and then re-examined in a multiplex-stained bladder cancer slide. FFPE tumor sections were stained on the BOND-RX autostainer (Leica Microsystems, Wetzlar, Germany), and staining conditions are described in Table 1. At the end of the staining protocols, slides were mounted with the ProLong Diamond antifade mountant (ThermoFisher Scientific, Waltham, MA, USA).

**Table 1:** mIF panels and staining conditions

	Antibody	Clone	Source	Dilution	Fluorophore
**1st panel**	CD4	4B12	ThermoFisher Scientific	1:10	Opal-650
	CD8	C8/144B	Agilent	1:200	Opal-480
	CD68	KP1	Agilent	1:100	Opal-540
	CD20	L26	Agilent	1:400	Opal-620
	FoxP3	D2W8E	Cell Signalling (Danvers, MA, USA)	1:50	Opal-570
	Granzyme B	11F1	Leica Biosystems	1:100	Opal-520
	CD163	10D6	Leica Biosystems	1:150	Opal-690
	Pan-cytokeratin (CK)	AE1/AE3	Agilent	1:100	Opal-780
	Spectral DAPI		Akoya Bioscences		
**2nd panel**	Neutrophil Elastase	NP57	Agilent	1:100	Opal-570
	CD44v3	VFF-327-v3	ThermoFisher Scientific	1:40	Opal-520
	CD44v6	VFF-7	ThermoFisher Scientific	1:200	Opal-480
	CD44v9	RV3	ThermoFisher Scientific	1:50	Opal-620
	CD56	123C3	Agilent	1:30	Opal-540
	CD44s	F10-44-2	Abcam (Cambridge, UK)	1:200	Opal-690
	Ki-67	MM-1	Leica Biosystems	RTU	Opal-650
	Pan-cytokeratin (CK)	AE1/AE3	Agilent	1:100	Opal-780
	Spectral DAPI		Akoya Bioscences		
**3rd panel**	ICAM-1 (CD54)	E3Q9N	Cell Signalling	1:150	Opal-540
	RHAMM (CD168)	EPR4055	Abcam	1:100	Opal-570
	Pan-cytokeratin (CK)	AE1/AE3	Agilent	1:100	Opal-780
	Spectral DAPI		Akoya Bioscences		

### Multispectral imaging

Multiplex-stained slides were acquired using the multispectral microscope Mantra Workstation 2.0 (Akoya Biosciences) at 20X magnification, considering only areas comprising tumor cells. The inForm Image Analysis software (Akoya Biosciences) was used to unmix and analyze multispectral images, and to create algorithms of analysis through its training with a selection of representative fields, as previously reported [9–12]. The pan-cytokeratin (CK) staining was used to differentiate infiltrating immune cells within the tumor areas and in the surrounding stroma in the tissue segmentation step. Then, single cells were segmented by nuclear counterstaining, and co-localized cell surface or intracellular markers were used to determine cell phenotypes. For the first, second and third panel we generated four, six and three different algorithms of analysis, respectively, and the relative algorithms were applied in the batch analysis of all acquired multispectral images of the same panel. Cell density data were calculated as the sum of the cells positive for a specific marker, divided by the area analyzed from the same tissue slide. Cell density and cell percentage results refer to the total area analyzed (tumor plus stroma), the intra-tumoral area only or the peri-tumoral stroma only, as indicated.

### Spatial metrics analyses

To assess the topological arrangement of immune cells in bladder cancer microenvironment and cell-to-cell interactions, spatial metrics between cells were calculated using phenoptrReports (add-ins for R Studio from Akoya Biosciences). In particular, the nearest neighbor analysis calculates the average distance between each feature’s centroid and its nearest neighbors’ centroid location, and it was used to analyse the mean distance between different cell subtypes. Moreover, the count within analysis was employed to calculate for each pair of phenotypes, the number of cells with a distinct phenotype having a cell of another phenotype within a specified radius. Since a distance radius of 20-25 μm between two cell subtypes is considered indicative of an enhanced probability for cell-to-cell contact [13], we calculated the number of reference cells that are present within a 20-25 μm radius from a cell with a different phenotype, and normalized for the total number of reference cells expressed as the percentage among the total number of reference cells. This methodology was used in order to not only consider the absolute number of cell-to-cell contacts, which might be viewed as an epiphenomenon of cellular density, but also to correct for the different number of immune cells present in the bladder TME.

### Gene expression analysis

Total RNA was extracted from 4 μm-thick FFPE tumor samples obtained before starting ONCOFID-P-B™ treatment, using the RNAesy FFPE kit (Qiagen, Hilden, Germany). RNA quantification was performed with Nanodrop 1000 spectrophotometer (ThermoFisher Scientific), and the RNA integrity and quality were evaluated with the Bioanalyzer 2100 (Agilent, Santa Clara, CA, USA). The PanCancer Immune Profiling panel (NanoString Technologies, Seattle, WA, USA) was used to measure the expression of 770 immune-related genes covering innate and adaptive immune responses [14]. The panel included 20 housekeeping genes, 8 negative controls and 6 synthetic positive controls. The samples were processed according to the manufacturer’s instructions and kits provided by NanoString Technologies. Sample RNA was hybridized with panel probes for 19 hours at 65 °C, and then complexes were processed on the nCounter FLEX platform (NanoString Technologies). Cartridges were scanned at 555 fields of view. Gene expression data were analyzed with the nSolver 4.0 Software (NanoString Technologies), and a quality check was performed. Raw data were normalized using a ratio of the expression value to the geometric mean of all housekeeping genes on the panel. Data were then Log2 transformed. The nCounter Advanced Analysis module V.2.0.134 software (NanoString Technologies) was used for differential expression analysis, and to obtain scores for cell type profiling and signature analysis, based on the expression of predefined genes.

### Analysis of bladder cancer dataset from the Cancer genome atlas (TCGA)

The bladder cancer (BLCA) dataset in the TCGA repository consists of 404 patient samples for which RNA-seq and clinical data are available [15]. Patients survival data were obtained from the supplementary material of Liu et al. [16], as suggested by Idogawa and colleagues [17]. The normalized expression of transcripts in the TCGA bladder cancer dataset was retrieved from the FIREBROWSE web utility (http://firebrowse.org/?cohort=BLCA) [18] and from the Broad Institute TCGA Genome Data Analysis Center (10.7908/C11G0KM9). To evaluate the intra-tumoral macrophage density, we applied the CIBERSORTx deconvolution method [19], which estimates the fraction of 22 immune cell types from the expression profiles of bulk RNA-seq samples, including three types of CD68+ macrophages (M0, M1, and M2). We computed gene expression profiles (GEPs) of the TCGA BLCA samples from the normalized expression of transcript isoforms data. The GEPs were uploaded to the CIBERSORTx online utility, and the resulting immune cell fractions were used to estimate macrophage infiltration. All the analyses were performed with the R programming language v4.3.2. Survival analysis was conducted using the Cox proportional hazards regression model implemented in the survival v3.5-7 R package. Further, the following packages were used: kableExtra v1.4.0, finalfit v1.0.7, riskRegression v2023.12.21, condsurv v1.0.0, tidycmprsk v1.0.0, gtsummary v1.7.2, ggsurvfit v1.0.0, lubridate v1.9.3, ggpubr v0.6.0, ggthemes v5.0.0, ggridges v0.5.5, ggplot2 v3.4.4.

### Statistical analysis

All statistical analyses were carried out using GraphPad Prism software (version 7.0) and IBM SPSS Statistics (version 28). Clinical data were correlated with cell densities, cell distribution and cell interactions analyzed at each time point. Non-parametric two-tailed Mann-Whitney test between two groups was used to compare the associations between variables. The Wilcoxon-rank sum test was used to compare the level of immune markers before and after the treatment. Statistical differences in HA receptors expression over time were determined with repeated measures 2-way ANOVA and Holm-Sidak multiple comparison post hoc test. To investigate association between immune factors and patient outcome, median values were used to dichotomize immune variables in subgroups; then, the Kaplan-Meier method was used to estimate survival curves, and the log-rank test was used to test difference between groups. Moreover, univariate Cox regression modelling for proportional hazards was used to calculate hazard ratio (HR) and 95% confidence interval (CI) for the association of dichotomized immune variables and patient outcome. For the correlation analyses, the non-parametric Spearman’s correlation coefficient (r) was calculated. Differences in gene expression between tumor samples from responding or not-responding patients were assessed using the t-test. The combined score of intra-tumoral macrophage density and CD44v6 expression was calculated from the estimated coefficient of each variable in a bivariate logistic model for complete response at the end of the treatment: intra-tumoral CD68 density (0 = low; 1 = high) *1.73 + CD44v6 expression (0 = low; 1 = high) *1.212. The performance of the combined score was estimated by determining the area under the receiving operator curve (AUC). All reported *p*-values are two-sided and *p* ≤ 0.05 was considered statistically significant.

## Results

### The immune TME composition differs between CR patients and non-responders (NR)

**Fig. 2:**
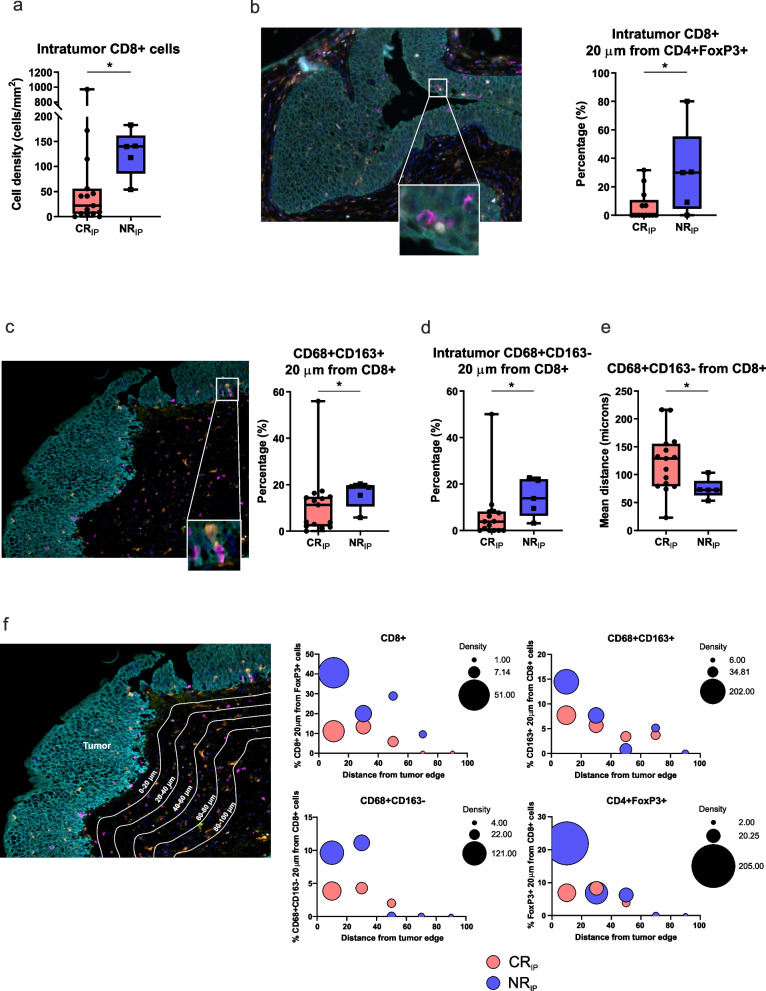
Characterization of the immune infiltrate in bladder CIS at baseline in patients achieving or not a CR after the intensive phase. **a** Intra-tumoral density (cells/mm^2^) of CD8+ T cells. **b** (Left) Representative image of a bladder CIS sample stained with the first mIF panel. In the crop, the proximity between CD8+ cells (magenta staining) and a CD4 + FoxP3+ cell (white and green staining) is highlighted. Original magnification × 20. (Right) Percentage of CD8+ T cells within a radius of 20 μm from CD4 + FoxP3+ Treg cells within the tumor regions. **c** (Left) Representative image of a bladder CIS sample stained with the first mIF panel. The color code is the same as in (b). In the crop, the proximity between a CD68 + CD163+ macrophage (grey and orange staining) and CD8+ cells (magenta staining) is highlighted. Original magnification × 20. (Right) Percentage of CD163+ M2-polarized macrophages within a radius of 20 μm from CD8+ T lymphocytes. **d** Percentage of CD68 + CD163- macrophages within a radius of 20 μm from CD8+ T lymphocytes within the tumor regions. **e** Mean distance (μm) between each CD68 + CD163- macrophage and the nearest CD8+ T lymphocyte. Significantly different data are represented by *p < 0.05. Floating box extends from 25th to 75th percentiles, line through the box indicates median, and bars extend from the smallest to largest values. **f** Schematic representation of the analysis of the number and type of cell interactions carried out progressively moving away from the tumor margin. Bubble graphs show the percentage of cell-to-cell interactions (dimension of the bubbles), progressively moving away from the tumor margin in CR_IP_ and NR_IP_ patients

We first compared the composition of immune TME at baseline, between patients achieving or not the CR after the IP (CR_IP_ and NR_IP_, respectively). The densities of CD4+ T cells, B lymphocytes (CD20+ cells) and tumor-associated macrophages (TAMs; CD68 + CD163- and CD68 + CD163+ cells) were comparable between the two patient groups, while natural killer cells (CD56+) and neutrophils (neutrophil elastase+) appeared negligible in all patients (Supplementary_Figure_1). Intriguingly, NR_IP_ exhibited a higher intra-tumoral infiltration of CD8+ cytotoxic T lymphocytes (CTLs) as compared to CR_IP_ patients (Fig. 2a). Notwithstanding, in NR_IP_ a higher percentage of such CTL were in proximity to CD4 + FoxP3+ T-regulatory cells (Treg; Fig. 2b) or TAMs (Fig. 2c-e). Additionally, the analysis of the number and type of cell interactions carried out progressively moving away from the tumor margin revealed that CR_IP_ patients had less CTLs in proximity to the tumor edge but their interactions with immunosuppressive subsets (Treg and TAMs) remained constantly low (Fig. 2f). Conversely, CTLs present in NR_IP_ within a distance ranging from 0 to 20 μm from tumor edge were more numerous but also closer to Treg or TAMs, these interactions progressively decreasing only moving away from the tumor edge (Fig. 2f).

**Fig. 3:**
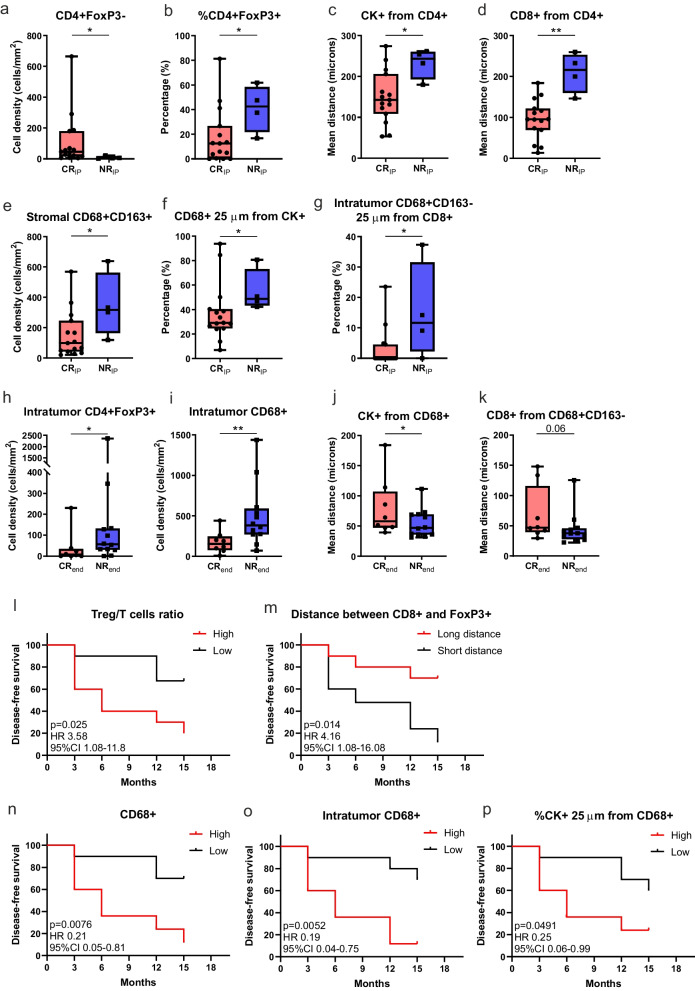
The impact of the TME contexture in patient response to ONCOFID-P-B™. **a-g** Characterization of the immune infiltrate in bladder CIS collected after the intensive phase in patients achieving or not a CR after the intensive phase. **h-k** Characterization of the immune infiltrate in bladder CIS collected at baseline in patients achieving or not a CR at the end of the 15-month study. Significantly different data are represented by *p < 0.05, **p < 0.01. Floating box extends from 25th to 75th percentiles, line through the box indicates median, and bars extend from the smallest to largest values. **l-p** Kaplan-Meier survival curves for disease-free survival according to the immune cell composition and cell-to-cell interactions at baseline in ONCOFID-B-P™-treated bladder CIS patients. The median cut-off of each immune variable was used to separate high and low infiltrated groups. Log-rank *p* values, hazard ratios (HR) and 95% confidence intervals (CI) are reported in each graph

In the biopsies collected after the IP, the TME of CR_IP_ patients resulted enriched in CD4 + FoxP3- T cells as compared to NR_IP_ (Fig. 3a,b). Moreover, the mean distance between CD4+ T lymphocytes and CK+ cells (Fig. 3c) or CD8+ T cells (Fig. 3d) was shorter in CR_IP_ patients. On the other hand, NR_IP_ presented more abundant CD68 + CD163+ TAMs in the stromal compartment (Fig. 3e), and closer interactions between macrophages and CK+ cells (Fig. 3f) or CTLs (Fig. 3g), as compared to CR_IP_ patients.

Finally, we compared the TME of the biopsies collected at baseline according to the clinical response reached at the end of the study (CR_end_ and NR_end_). In this case, both intra-tumoral Treg and macrophages were more abundant in NR_end_ samples (Fig. 3h,i), with macrophages being closer to tumor cells (Fig. 3j) and CTLs (Fig. 3k).

The higher Treg/T cell ratio and the shorter mean distance between CTLs and Treg were associated with a shorter DFS (Fig. 3l,m). Furthermore, a longer DFS was associated with an overall lower density of macrophages (Fig. 3n) and, in particular, of intra-tumoral TAMs (Fig. 3o). Moreover, a higher percentage of tumor cells in close proximity to macrophages was associated with a shorter DFS (Fig. 3p). Collectively, these observations suggest that, rather than the mere presence of CD8+ T cells within the TME, are the interactions between such T lymphocytes and immunosuppressive cells that limit their anti-tumoral activity, to play a key role in bladder CIS progression. Moreover, we identified a key negative predictive role for Treg and TAMs in the patient response to ONCOFID-P-B™.

### HA receptors expression differs between responding and non-responding patients

**Fig. 4:**
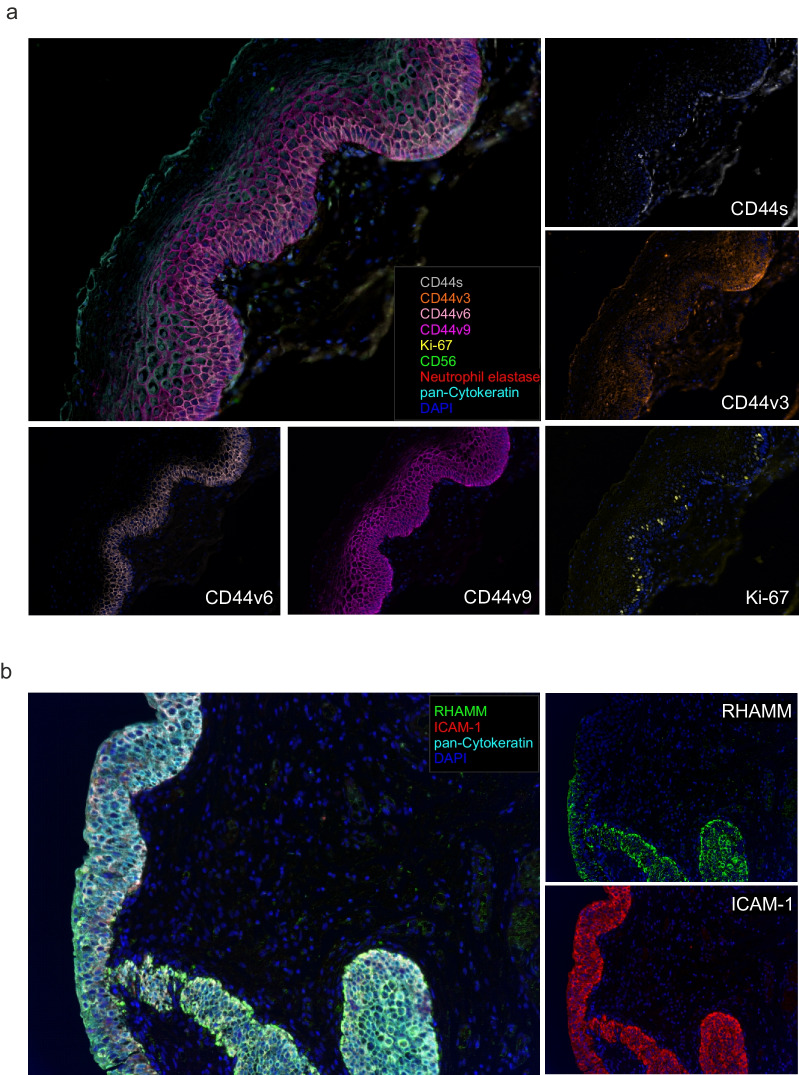
Assessment of HA receptors expression and distribution in bladder CIS samples. **a** Representative 9-color multispectral image of the second mIF panel. Markers and color codes are indicated in the figure. Single markers assessment of the CD44 variants is depicted around the merged image. **b** Representative 4-color multispectral image of the third mIF panel. Markers and color codes are indicated in the figure. Single markers assessment of the ICAM-1 and RHAMM is depicted on the right of the merged image. Original magnification 20x

We examined the expression and distribution of the principal HA receptors, namely the CD44 as the standard isoform (CD44s) and its most represented variants (CD44v3, CD44v6, CD44v9), ICAM-1 and RHAMM (Fig. 4). The staining revealed differential expression patterns among HA receptors in bladder CIS: CD44s was preferentially expressed in the basal urothelial cell layer and lamina propria, CD44v6 in the basal urothelial cell layer, while CD44v3 and CD44v9 were strongly evidenced in the basal and intermediate urothelial cell layers (Fig. 4a). ICAM-1 and RHAMM were distributed throughout the urothelium (Fig. 4b).

**Fig. 5:**
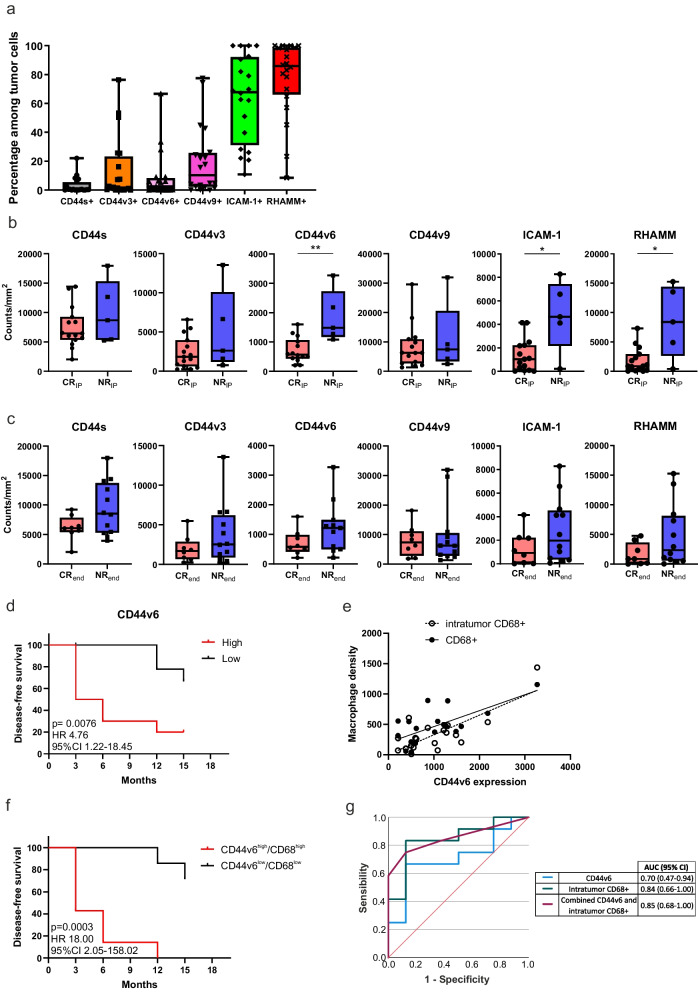
Correlation between HA receptors expression at baseline and patient response to ONCOFID-P-B™. **a** Percentage of tumor cells expressing each HA receptor at baseline. **b-c** Expression (counts/mm^2^) of each HA receptor at baseline according to clinical response **(b)** after the 12-weekly ONCOFID-P-B™ instillation of the intensive phase and **(c)** at the end of the 15-month study. Significantly different data are represented by *p < 0.05, **p < 0.01. Floating box extends from 25th to 75th percentiles, line through the box indicates median, and bars extend from the smallest to largest values. **d** Kaplan-Meier survival curves for disease-free survival according to the expression of CD44v6 at baseline in ONCOFID-B-P™-treated bladder CIS patients. The median cut-off of each variable was used to separate high and low groups. Log-rank p values, hazard ratios (HR) and 95% confidence intervals (CI) are reported in the graph. **e** Correlation between CD44v6 expression and the density of total (Spearman r = 0.5193 95% CI 0.07 to 0.79; *p* = 0.022) or intra-tumoral CD68+ macrophages (r = 0.54 95% CI 0.09 to 0.80; *p* = 0.016). **f** Kaplan-Meier curves for disease-free survival stratifying patients according to the expression of CD44v6 and the intra-tumoral CD68 density. The median value of each variable was used as cut-off to identify high and low subgroups. Log-rank p values, hazard ratios (HR) and 95% confidence intervals (CI) are reported in each graph. **g** Receiving Operator Curve (ROC) showing the performance of the combined CD44v6 expression and intra-tumoral CD68+ cells to predict patient response to ONCOFID-P-B™

In all baseline biopsies we found the expression of at least one CD44 isoform on tumor tissue, with the different variants being often co-expressed by cancer cells and CD44v9 the most represented (Fig. 5a). ICAM-1 and RHAMM were expressed in the majority of tumor cells (Fig. 5a). Only a small proportion of CD44 isoform-expressing cancer cells was also positive for the Ki-67 proliferation marker (Supplementary_Figure_2a).

Among the CD44 isoforms considered independently, NR_IP_ turned out to express significantly higher levels of CD44v6 as compared to CR_IP_ patients (Fig. 5b). Moreover, NR_IP_ showed a higher expression of both ICAM-1 and RHAMM, as compared to CR_IP_ patients (Fig. 5b). This trend was maintained in CR_end_ and NR_end_ patients (Fig. 5c). Accordingly, the only variable with a predictive value was CD44v6, as patients with a high CD44v6 expression had a shorter DFS as compared to patients with a low expression of the isoform (Fig. 5d and Supplementary_Figure_2b).

Moreover, a direct correlation existed between CD44v6 expression and the density of CD68+ macrophages (Spearman r = 0.5193 95% CI 0.07 to 0.79; *p* = 0.022), and in particular with intra-tumoral macrophages (Spearman r = 0.54 95% CI 0.09 to 0.80; *p* = 0.016; Fig. 5e). Thus, patients were divided in two groups (CD44v6^low^/CD68^low^ versus CD44v6^high^/CD68^high^) depending on CD44v6 expression level and intra-tumoral CD68+ macrophages density, to be thereafter correlated with DFS. CD44v6^high^/CD68^high^ patients had a significantly worst prognosis as compared to CD44v6^low^/CD68^low^ patients (Fig. 5f). The derived integrated score had an AUC of 0.85 (95%CI 0.68–1.00) for patient response prediction (Fig. 5g). Therefore, the combined evaluation of both CD44v6 expression and intra-tumoral macrophage density provided a biomarker with increased predictive value for patient response to ONCOFID-P-B™.

### ONCOFID-P-B™ modulates immune cell populations and HA receptors expression

**Fig. 6:**
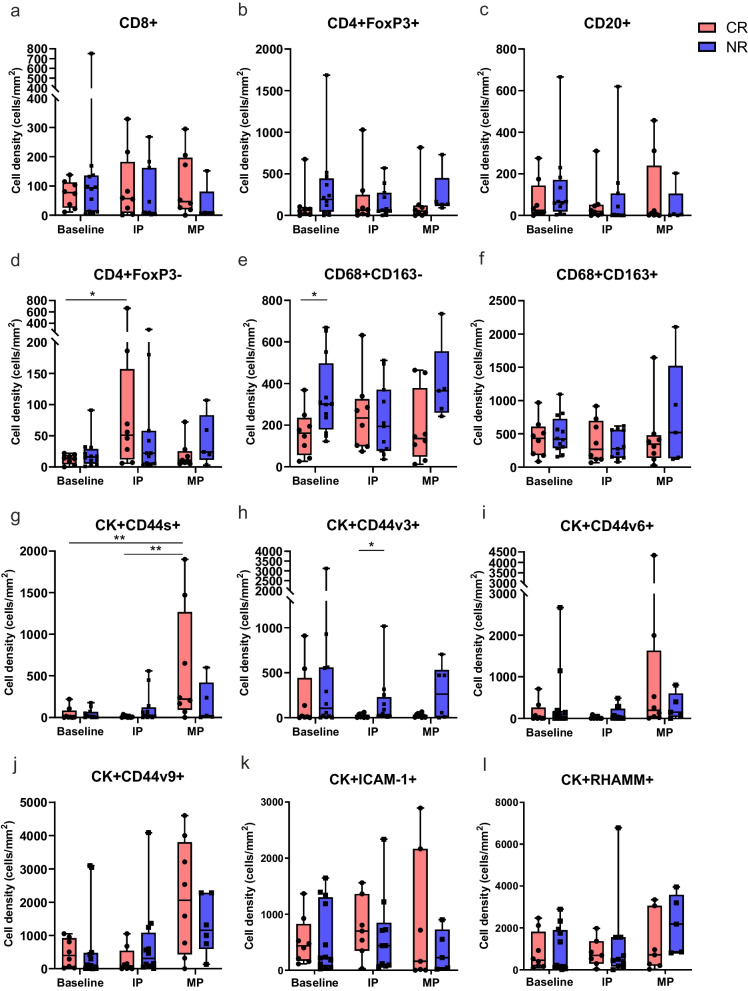
Changes in immune subsets and HA receptors expression on tumor cells induced by ONCOFID-P-B™ treatment. Density (cells/mm^2^) of (**a-f**) immune cell populations and (**g-l**) tumor cells expressing each HA receptor at baseline, after the intensive phase (IP) and during or after the maintenance phase (MP) in responding and non-responding patients. Significantly different data are represented by *p < 0.05 and **p < 0.01. Floating box extends from 25th to 75th percentiles, line through the box indicates median, and bars extend from the smallest to largest values

We then analyzed the changes in immune subsets and HA receptors expression on tumor cells induced by ONCOFID-P-B™ treatment. CTLs, Treg and B lymphocytes densities were minimally modified by ONCOFID-P-B™ treatment both in CR_end_ and NR_end_ patients (Fig. 6a-c). On the other hand, CD4 + FoxP3- T cells increased after the IP only in responding patients, to return thereafter to the basal level after the MP (Fig. 6d). Conversely, while ONCOFID-P-B™ treatment induced very limited variations in macrophage densities, we observed a trend for an increase in TAMs when patients relapsed during the MP (NR_MP_) (Fig. 6e,f).

Regarding HA receptors expression, all CD44 isoforms were affected by the treatment in either patient groups, likely a feature reflecting a direct interaction between ONCOFID-P-B™ and HA receptors. Indeed, the density of tumor cells expressing CD44s appeared significantly increased in CR_end_ patients after MP (Fig. 6g), while CD44v3 progressively decreased throughout the treatment protocol (Fig. 6h). Moreover, CD44v6 and CD44v9 in either patient groups appeared increased at the end of the treatment, albeit not significantly (Fig. 6i,j). Differently, the changes in ICAM-1 and RHAMM expression induced by ONCOFID-P-B™ were very limited both in responding and non-responding patients (Fig. 6k,l).

### In CR_end_ patients, a normal bladder immune contexture is re-established after the maintenance phase

**Fig. 7:**
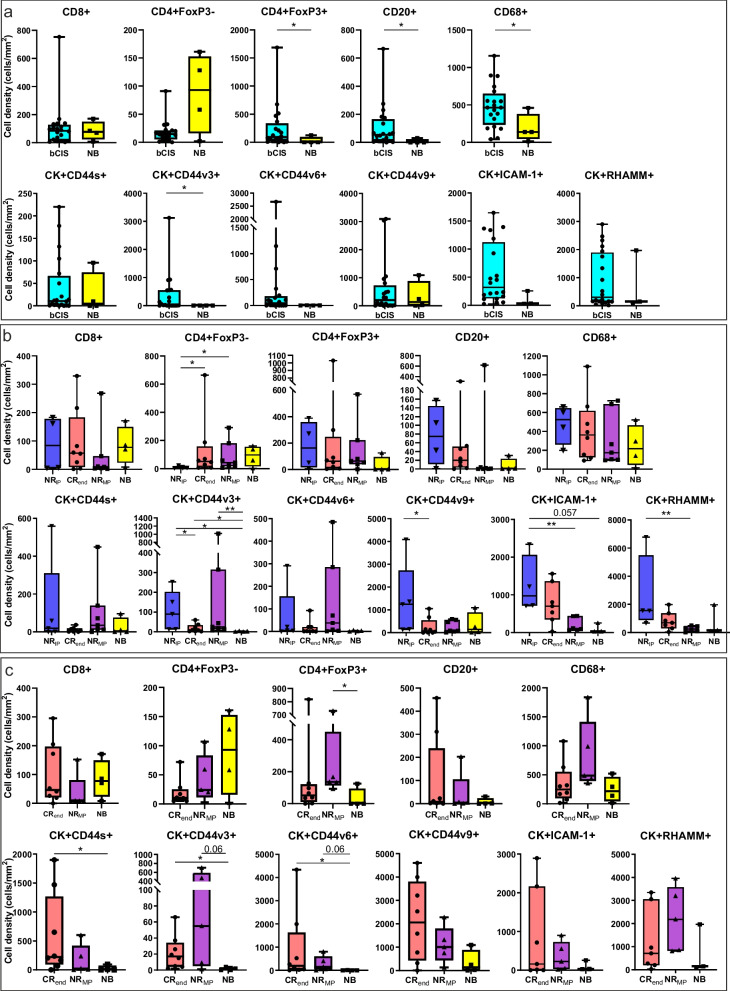
Comparison of immune TME and HA receptors expression between bladder CIS and normal bladder samples. **a** Density (cells/mm2) of immune cell populations and tumor cells expressing each HA receptors in bladder CIS collected at baseline and normal bladders. **b** Density (cells/mm2) of immune cell populations and tumor cells expressing each HA receptors in bladder CIS samples collected after the IP and in normal bladders. Bladder CIS patients were grouped according to the clinical response (NR_IP_: patients who did not respond to ONCOFID-P-B™ treatment after the intensive phase; CR_end_: patients with a complete pathological response at the end of the 15-month study; NR_MP_: patients who relapsed during the maintenance phase). **c** Density (cells/mm2) of immune cell populations and tumor cells expressing each HA receptors in bladder CIS samples collected after the MP and in normal bladders. Bladder CIS patients were grouped according to the clinical response (CR_end_: patients with a complete pathological response at the end of the 15-month study; NR_MP_: patients who relapsed during the maintenance phase). Significantly different data are represented by *p < 0.05 and **p < 0.01. Floating box extends from 25th to 75th percentiles, line through the box indicates median, and bars extend from the smallest to largest values

Four normal bladder samples were also collected, stained and analyzed to compare their immune infiltrate and HA receptors expression pattern with those observed in bladder CIS samples. Baseline bladder CIS specimens had a higher density of Treg, B lymphocytes and macrophages as compared to normal bladder (Fig. 7a). Normal epithelial cells stained moderately for CD44v9 and RHAMM, low for CD44s and ICAM-1, and negligibly for CD44v3 and CD44v6 isoforms (Fig. 7a).

The immune contexture of bladder CIS samples collected after the IP appeared similar to what observed in NB (Fig. 7b). Similarly, in CR_end_ and NR_MP_ patients, the differences in the expression of HA receptors tended to smooth as compared to normal bladders, with the exception of CD44v3 that remained still elevated (Fig. 7b). However, in NR_IP_ the expression of CD44v3, CD44v9, ICAM-1 and RHAMM was more elevated as compared to CR_end_ patients and normal bladders (Fig. 7b).

Finally, in biopsies collected during or after the MP, the density of infiltrating immune cells in CR_end_ patients was comparable to normal bladders (Fig. 7c). Conversely, in NR_MP_, Treg and macrophages resulted still elevated as compared to normal bladders (Fig. 7c). Moreover, we observed a trend for a higher expression of HA receptors in CR_end_ and NR_MP_ patients as compared to normal bladders (Fig. 7c).

### Gene-based cell types and signatures are differentially expressed between CR_end_ and NR_end_ patients, and between patients with high or low CD44v6 expression

We investigated potential differences in gene expression in baseline biopsies between CR_end_ and NR_end_ patients. Due to the very limited tumor tissues available, only 3 CR_end_ and 3 NR_end_ successfully passed the quality controls, and therefore were considered for the subsequent gene expression analysis. In CR_end_ patients, a trend for a higher expression of ALCAM, ITGAE and CXCL16 genes was observed (Supplementary_Figure_3a). Based on the expression of cell-type and signature-associated predefined genes present in the panel, we found a trend for a higher expression of genes associated to CD8 T cells, Th1 cells, Treg cells, Exhausted CD8 cells and macrophages in NR_end_ patients as compared to CR_end_ (Supplementary_Figure_3b). Moreover, several gene signatures were found differentially regulated between CR_end_ and NR_end_ patients (Supplementary_Figure_3c). To validate these results, the TME of these selected patients was analysed in terms of immune cell infiltration and spatial distribution, and HA receptors expression. A trend for a higher infiltration of Treg cells and TAMs was found in NR_end_ patients as compared to CR_end_ (Supplementary_Figure_3d), as well as a higher percentage of CD8+ T cells in close proximity to Treg cells and TAMs (Supplementary_Figure_3e). Moreover, NR_end_ patients disclosed a trend for a higher expression of HA receptors except for CD44v9 (Supplementary_Figure_3f).

In addition, we stratified the 6 patients according to the expression of CD44v6 (higher or lower than the median), and found that patients with a higher expression of CD44v6 had a lower ratio between TILs-related and exhausted CD8-related genes (Supplementary_Figure_3g). Moreover, in patients with higher CD44v6 levels, genes related with regulation, chemokines, macrophage functions and T cell functions were overexpressed as compared to patients with lower CD44v6 levels. Conversely, genes related to transporter functions, tumor-inflammation signature, cytotoxicity, antigen processing and adhesion were downregulated in patients with higher expression of CD44v6 (Supplementary_Figure_3h).

The combined evaluation of CD44v6 coding transcript and estimated macrophage infiltration is an independent prognostic biomarker in the TCGA bladder cancer cohort.

**Fig. 8:**
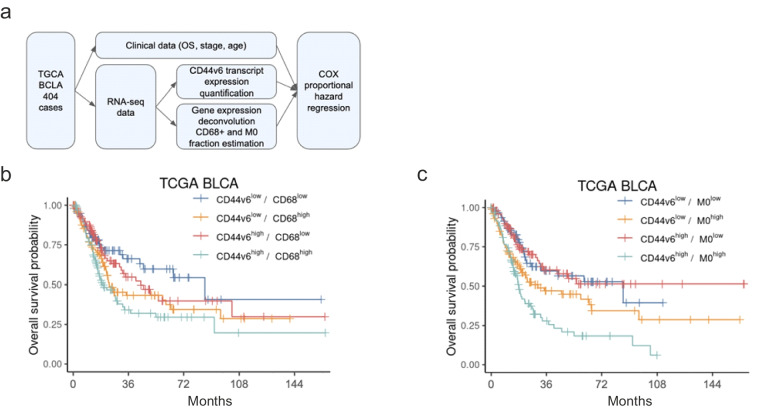
The prognostic value of CD44v6 transcript and estimated intra-tumoral macrophages in the TCGA Bladder Cancer (BLCA) cohort. **a** Flowchart of the analysis performed on the TCGA BLCA clinical and transcript expression data. **b**, **c** Kaplan-Meier survival curves for overall survival of the TCGA BLCA samples considering combined expression levels of CD44v6 transcript and **b**) estimated total fraction of CD68+ cells or **c**) the M0 macrophage fraction. High and low levels were computed according to the median in the dataset

We considered the whole TCGA BLCA dataset that includes both clinical and RNA-seq data of 404 patients, to correlate the expression of the transcript encoding the CD44v6 isoform and the CD68+ cell fraction in tumor samples with clinical features of patients (Fig. 8a). Of note, the bulk RNA-seq sample deconvolution analysis using CIBERSORTx allowed to estimate the fraction of 22 immune cell types, including three subsets of CD68+ macrophages (M0, M1, and M2).

In the TCGA cohort, stage I tumors were quite rare (5 cases, 1.2%) and therefore we considered grouping with stage II cancers (overall 131 cases, 32.4%), while stages III and IV accounted for 34.7 and 32.9%, respectively (Supplementary_Table_2).

Patient stratification according to the median expression value of the CD44v6 isoform-coding transcript, disclosed that the high expression of CD44v6 was associated with an increased risk (HR 1.22, 95% CI 0.91-1.65, *p* = 0.179; Supplementary_Table_2). Moreover, we stratified patients according to the median value of all estimated CD68+ macrophages in the tumor samples (M0 + M1 + M2). In univariate analysis, CD68^high^ cases had a significantly worse prognosis as compared to CD68^low^ patients (HR 1.61, 95% CI 1.19-2.18, *p* = 0.002) (Supplementary_Table_2). Notably, the combination of the two factors evidenced that patients with CD44v6^high^/CD68^high^ had a significantly worse prognosis than CD44v6^low^/CD68^low^ patients (HR 2.02, 95% CI 1.30-3.15, p = 0.002; Fig. 8b and Supplementary_Table_2), with a median survival of 19.4 months versus 86.8 months, respectively. The prognostic value of the combination remained significant in multivariate analysis that also considered tumor stage and patient age (HR 1.85, range 1.18-2.89, *p* = 0.007), both significant predictors of outcome [20] (Supplementary_Table_2 and Supplementary_Figure_4).

Since the deconvolution analysis allowed to estimate the fraction of three distinct macrophage populations (M0, M1 and M2), we investigated the prognostic role of such subtypes more in detail. We observed that the M0 population was linked with an increased risk (HR = 2.07, 95% CI 1.52-2.82, *p* < 0.001), whereas the M1 and the rarest M2 macrophage fractions did not have prognostic relevance (Supplementary_Table_3). Combining the evaluation of both CD44v6 transcript expression and M0 macrophages, patients with CD44v6^high^/M0^high^ had a significantly increased risk than patients with CD44v6^low^/M0^low^ (HR 2.53, 95% CI 1.64-3.92, p < 0.001; Fig. 8c and Supplementary_Table_3), which was even higher than considering the CD68+ cells altogether. This risk remained significantly higher also in multivariate analysis with age and stage (2.21, 95% CI 1.42-3.46, p < 0.001) (Supplementary_Table_3 and Supplementary_Figure_5).

## Discussion

The treatment strategy with ONCOFID-P-B™ relies on the interaction of HA with its receptors, followed by the internalization of the conjugate within tumor cells with the subsequent release of paclitaxel in its active form. In this regard, CD44 represents an HA receptor therapeutically interesting since it is known to be upregulated in cancer-initiating or metastasizing cells, and involved in the epithelial-mesenchymal transition (EMT), cancer cell survival and drug resistance [21, 22]. However, this rational approach to CD44 targeting by HA may be complicated by i) the alternative splicing of CD44 that leads to multiple variants with different affinity for HA, and responsible for different signalling pathways [5], and ii) the unclear relation between CD44 expression, HA binding and internalization [23, 24].

Currently, the role of CD44 in tumor-related clinical outcomes is still contradictory [25–29], and therefore further investigations are required to fully clarify the specific role of different CD44v in patient prognosis. Indeed, CD44 functions are regulated by a delicate balance of different factors, such as a minimal degree of glycosylation and optimal density values, above which the internalization process may slow down [30]. Moreover, HA internalization is a complex phenomenon of endocytic recognition likely mediated by protein complexes formation [31]. In this regard, we identified CD44v6 expression in bladder CIS patients at baseline as a negative predictive factor for response to ONCOFID-P-B™ treatment, in line with the observation that HT-29 tumor cells, which highly express the CD44v6 isoform, have a poorer internalization ability [23]. This is likely due to the formation of protein complexes with c-Met and Hepatocyte Growth Factor, which reduce CD44v6 endocytic performance while activating a signalling pathway leading to cancer invasiveness and metastatic spread [32, 33]. Moreover, CD44v6 is considered a critical marker of cancer-initiating/stem cells, as its role in niche formation [34], apoptosis resistance [35], EMT [36], and tumor progression and metastatic invasion [37]. Accordingly, a negative prognostic role for the CD44v6 isoform has been described for bladder, lung, breast, gastric and colon cancers [38–42]. All these features suggest that CD44v6-expressing bladder CIS may be intrinsically more aggressive and less susceptible to ONCOFID-P-B™ because of a reduced uptake of the conjugate.

Beyond HA receptors expression, the crosstalk between immune components and neoplastic cells is crucial for tumor progression. We report a negative predictive role for intra-tumoral TAMs in response to ONCOFID-P-B™ treatment, and a positive correlation between CD44v6 expression and intra-tumoral macrophages density. Accordingly, the combined evaluation of CD44v6 expression and intra-tumoral TAMs revealed a stronger predictive value for the stratification of patients with a high risk of recurrence. These observations are in line with Rao et al, who uncovered a reciprocal interaction between TAMs and CD44-positive colorectal cancer (CRC) cells during tumorigenesis [43]. Indeed, CD44-positive cells were found to promote the secretion of high level of osteopontin (OPN) by macrophages, which in turn binds to CD44 expressed by tumor cells promoting clonal growth via the activation of the JNK pathway, invasion and metastasis. Moreover, authors also showed that the combination of OPN and CD44v6 transcripts negatively correlated with CRC patient survival. Accordingly, the interaction between macrophage-secreted OPN and CD44v6 has been reported to drive cancer progression and metastasis in several cancers [44–46]. Moreover, OPN promotes stem cell-like proprieties and radiation resistance in adjacent tumor cells via activation of CD44 signalling [47]. Thus, perturbing the OPN–CD44 axis has been proposed as a therapeutic strategy to treat patients with metastatic bladder cancer [48]. Additionally, myeloid- and tumor cell–released OPN acts as an immune checkpoint to suppress CTL activation, and confers host tumor immune tolerance and immune evasion [49]. All these data can explain the apparently contradictory result that non-responders have more intra-tumoral CTL as compared to responders. In this regard, Baras et al. observed that a favorable association between the level of CD8+ T cells and the outcome of patients with bladder cancer can depend on the presence of other immune cell populations, including FoxP3+ Tregs cells [50]. Accordingly, we found that a higher fraction of CD8+ T cells in NR patients are closer to TAMs and FoxP3+ cells, making us to assume that macrophages and Treg act as immunosuppressive populations limiting CTL anti-tumor activity and patient response to ONCOFID-P-B™.

Additionally, we demonstrated that the combined evaluation of CD44v6 coding transcript and macrophages, in particular the M0 subtype, has a prognostic value also in the TCGA BLCA dataset. In ovarian cancer and glioblastoma, transcriptomic and proteomic profiling demonstrated that M0 macrophages disclose high expression of M2 markers and a transcriptional profile more similar to M2 macrophages [51, 52]. Moreover, M0 macrophages have been found to be one of the cell subsets most strongly associated with poor outcome in breast cancer [53], prostate cancer [54], lung adenocarcinoma [55], and bladder cancer [56, 57]. In addition, Wei and colleagues reported that M0 macrophages secrete OPN, which acts as a chemokine for pro-tumoral monocytes and macrophages (i.e. M0 and M2) in glioblastoma [58]. Notably, almost all of patients included in the TCGA BLCA dataset had tumor stages higher than stage I; therefore, results from this analysis support the concept that the combined evaluation of macrophages plus CD44v6 isoform can be adopted as prognostic biomarker also in urothelial cancer cohorts with advanced tumor stages, strengthening the potential of our findings.

Collectively, our results suggest that the complex reciprocal interactions between HA receptors on tumor cells, immunosuppressive cells/molecules and CTL infiltrating the TME play a key role in the clinical response to ONCOFID-P-B™ in bladder CIS patients. On the other hand, we also analyzed the variations induced by ONCOFID-P-B™ in the TME, taking into consideration that our patient cohort was not treatment-naïve but had already received BCG within 6 months from ONCOFID-P-B™ therapy start. Indeed, the effects of BCG on TME were likely still appreciable in the baseline bladder CIS, since they appeared more infiltrated as compared to normal bladder samples. In this regard, it has been reported that BCG therapy exerts pleiotropic effects, among which also the enhancement of the effector functions of tumor-specific CD4+ T cells [59]. Interestingly, CD4+ T lymphocytes further increased and were closer to epithelial or CD8+ T cells in CR patients after the IP, all signs of an activated and tumor-specific immune response that could have been directly fostered by ONCOFID-P-B™ action, as the HA mojety has intrinsic immunomodulatory effects. Similarly, Kates et al. reported a more pronounced infiltration of antitumor immunophenotypes in two BCG-naïve non-muscle invasive bladder cancer patients responding to a microparticle docetaxel experimental drug [60].

Like other cancers associated with long-term carcinogenic exposure, such as non-small cell lung cancer and melanoma, urothelial bladder cancer has been known to harbor relatively high tumor mutational burden (TMB) [61]. High TMB is associated with benefit from immunotherapy with BCG in non-muscle invasive bladder cancer [62]. Moreover, urothelial carcinomas with high TMB exhibit several molecular defects that could be exploited for combinatorial treatments [63]. Results from a meta-analysis interrogating dataset of 33 cancer types from TCGA, revealed that CD44 expression is negatively associated with TMB in bladder cancer [64]. In light of these observations, while it would have been interesting to include such information in our work, this genomic analysis was precluded by the paucity of the available biopsy materials. Notwithstanding, such limitations could be prospectively overcome by analysing the samples from an ongoing phase III, single-arm clinical study aimed to evaluate the efficacy and safety of ONCOFID-P-B™ administered intravesically to patients with BCG-unresponsive CIS of the bladder with or without Ta-T1 papillary disease (NCT05024773).

Overall, our data highlight the powerful intrinsic activity of ONCOFID-P-B™ and allow to advance a potential rationale for combinatorial treatments with different drugs: i) targeting the macrophage-CD44 axis or depleting the macrophage/Treg compartment could increase tumor surveillance by CD8+ T cells and make bladder CIS more responsive to ONCOFID-P-B™ treatment. ii) The combined instillation of both BCG and ONCOFID-P-B™ in bladder CIS patients might results in a synergic effect improving clinical activity because HA binding by effector T cells may help their recruitment to inflammatory sites and may improve their survival and function [65]. iii) Since PD-1/PD-L1 checkpoint expression has been reported to increase in BCG-resistant patients [66], a checkpoint blockade therapy could remove the immunosuppressive constrains in the TME and allow ONCOFID-P-B™ to be effective even in NR patients.

## Conclusions

In conclusion, we advance that a thorough analysis of both HA receptors and immune TME can provide more informative hints to predict bladder CIS response to ONCOFID-P-B™. This is in particular exemplified by the combined evaluation of intra-tumoral macrophages density and CD44v6 expression, a potentially new biomarker that showed high sensitivity and specificity for response prediction, and that can be easily reproduced by classical immunohistochemistry in the clinical setting. Although the combined score we advance appears promising, we are aware that it would require further validation in a larger cohort of patients, like those enrolled in the currently ongoing phase III, single-arm NCT05024773 clinical study.

### Supplementary Information

**Supplementary Material 1.**

## Data Availability

The data that support the findings of this study are available from Fidia Farmaceutici SpA but restrictions apply to the availability of these data, which were used under license for the current study, and so are not publicly available. Data are however available from the authors upon reasonable request and with permission of Fidia Farmaceutici SpA.

## Abbreviations

CIS: Carcinoma in situ
BCG: Bacillus Calmette-Guerin
PTX: Paclitaxel
HA: Hyaluronic acid
CD44v: CD44 variants
CR: Complete response
IP: Intensive phase
MP: Maintenance phase
TME: Tumor microenvironment
NR: Non-responders
DFS: Disease-free survival
mIF: Multiplex Immunofluorescence
FFPE: Formalin-fixed paraffin-embedded
CK: Pan-cytokeratin
HR: Hazard ratio
CI: Confidence interval
AUC: Area under the receiving operator curve
NR: Non-responders
TAMs: Tumor-associated macrophages
CTLs: Cytotoxic T lymphocytes
Tregs: T-regulatory cells
EMT: Epithelial-mesenchymal transition
CRC: Colorectal cancer
OPN: Osteopontin
TCGA: Bladder Cancer dataset
GEP: Gene expression profiling
TMB: Tumor mutational burden
