# Supplementary material for: A paclitaxel-hyaluronan conjugate (ONCOFID-P-B™) in patients with BCG-unresponsive carcinoma in situ of the bladder: a dynamic assessment of the tumor microenvironment
Source: J Exp Clin Cancer Res. 2024 Apr 10;43:109. doi: 10.1186/s13046-024-03028-5 (PMC11005197; doi:10.1186/s13046-024-03028-5)
Supplement: Supplementary file 1 — Supplementary Material 1. [file 13046_2024_3028_MOESM1_ESM.docx]

**Supplementary_Table_1. Normal bladder donors characteristics**

| **Patient ID** | **Age (years)** | **Sex** | **Cause of death** |
| --- | --- | --- | --- |
| #1 | 38 | Male | Cardio-respiratory failure |
| #2 | 69 | Male | Ischaemic heart disease |
| #3 | 44 | Female | Cardio-respiratory failure |
| #4 | 83 | Male | Ischaemic heart disease |


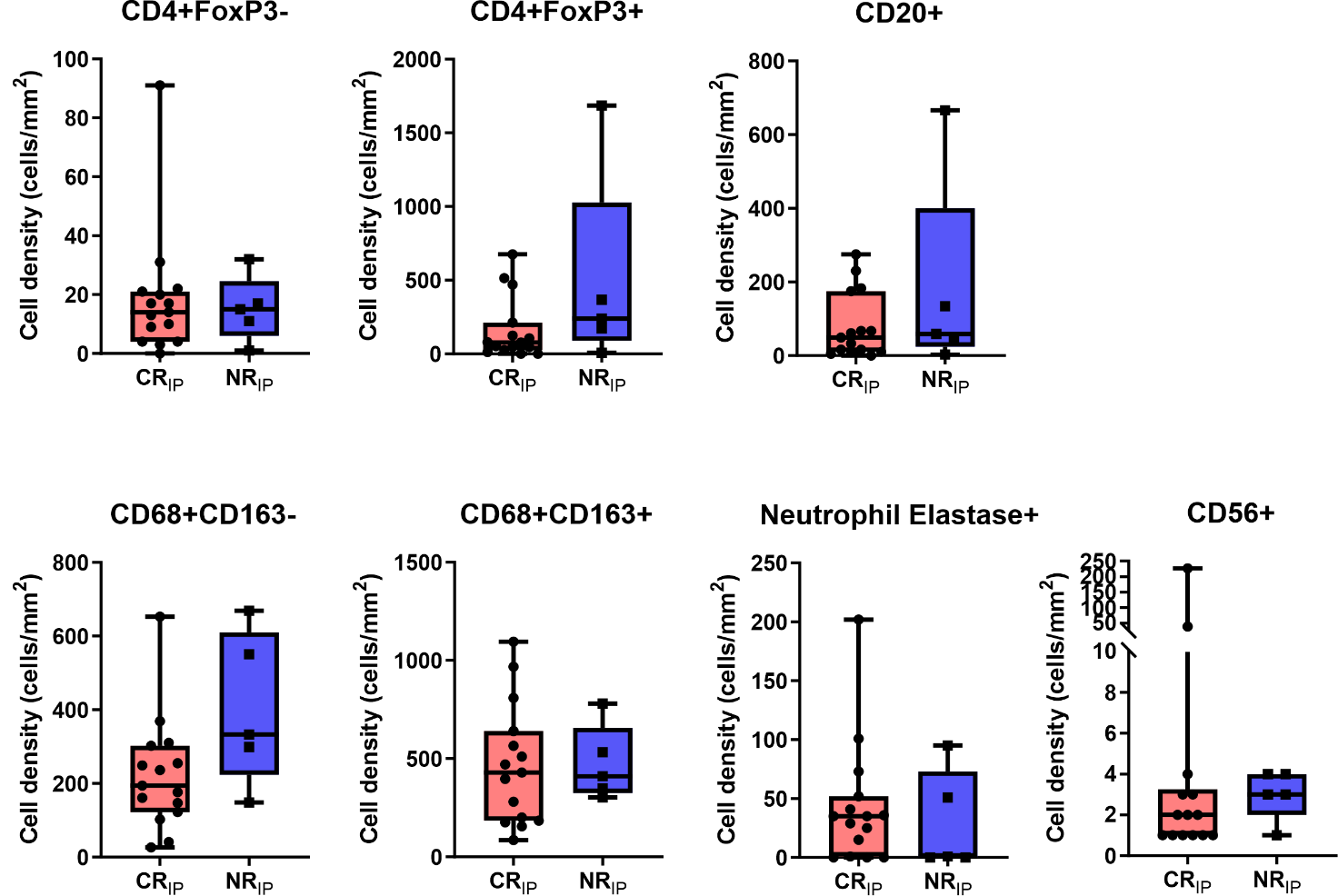


**Supplementary_Figure_1.** Density (cells/mm^2^) of immune cell populations infiltrating the bladder CIS microenvironment. Neutrophil elastase and CD56 staining identified neutrophils and NK cells, respectively. Floating box extends from 25th to 75th percentiles, line through the box indicates median, and bars extend from the smallest to largest values.


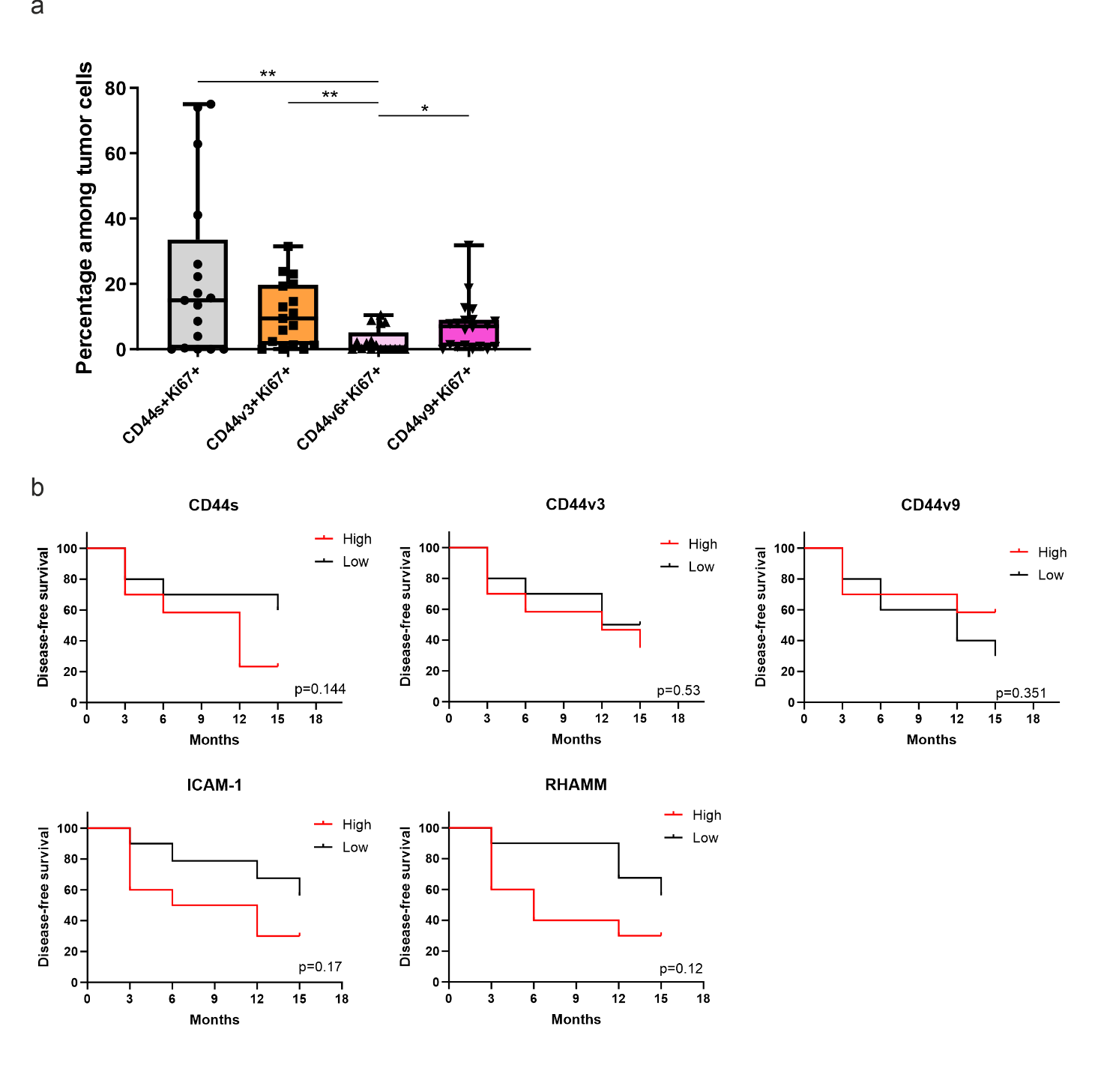


**Supplementary_Figure_2. a)** Percentage of Ki-67+ tumor cells expressing each CD44 isoforms at baseline. Floating box extends from 25th to 75th percentiles, line through the box indicates median, and bars extend from the smallest to largest values. **b)** Kaplan-Meier curves for disease-free survival according to the expression of HA receptors at baseline in ONCOFID-P-B™-treated bladder CIS patients. The median cut-off of each variable was used to separate high and low groups. Log-rank p values are reported in each graph.


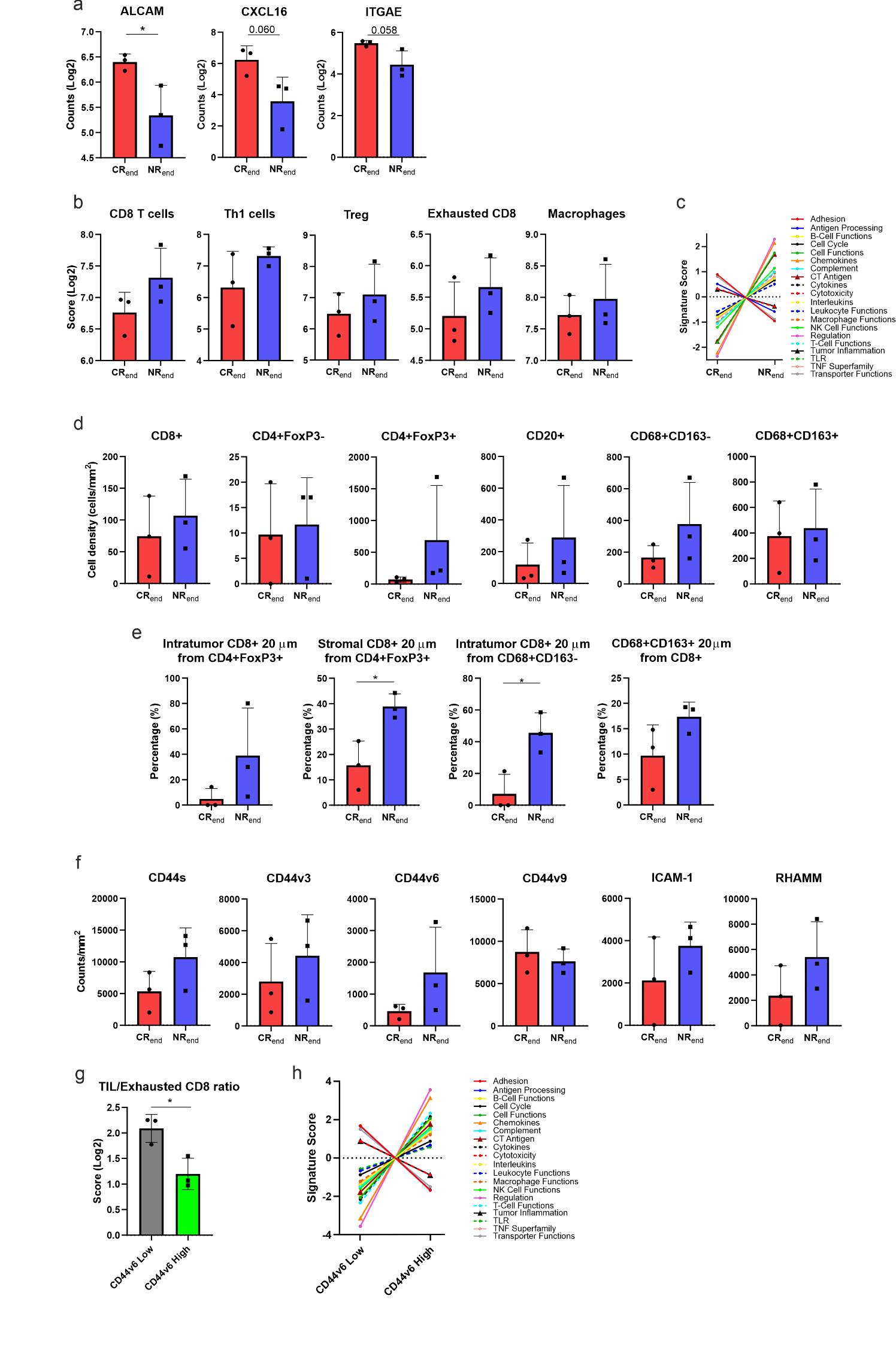


**Supplementary_Figure_3.** **Gene expression analysis. a)** Differential gene expression in bladder CIS at baseline in responding and non-responding patients. **b)** Differential expression of gene-based cell types and signatures in CR_end_ and NR_end_ patients. **c)** TME characterization of CR_end_ and NR_end_ patients for whom the gene expression analysis was performed. **d)** Differential expression of gene-based cell types and signatures in patients with expression of CD44v6 higher or lower than the median. Significantly different data are represented by *p <0.05.

|  |
| --- |

**Supplementary_Table_2. Cox-regression hazards ratio (HR) in univariate and multivariate analyses.**

| **Variable** |  | **Patients (percentage)** | **Univariate analysis** | **Multivariate analysis** |
| --- | --- | --- | --- | --- |
|  |  |  | **HR (95% CI, p-value)** | **HR (95% CI, p-value)** |
| **Age** | <65 years | 150 (37.1) |  |  |
|  | 65-74 years | 124 (30.7) | 1.90 (1.30-2.78, p=0.001) | 2.10 (1.43-3.08, p<0.001) |
|  | 75+ years | 130 (32.2) | 2.04 (1.39-2.97, p<0.001) | 1.93 (1.31-2.83, p=0.001) |
| **Tumor stage** | Stage I, II | 131 (32.4) |  |  |
|  | Stage III | 140 (34.7) | 1.61 (1.06-2.46, p=0.027) | 1.44 (0.94-2.20, p=0.094) |
|  | Stage IV | 133 (32.9) | 2.91 (1.96-4.32, p<0.001) | 2.79 (1.87-4.16, p<0.001) |
| **CD44v6** | CD44v6^low^ | 202 (50.0) |  |  |
|  | CD44v6^high^ | 202 (50.0) | 1.22 (0.91-1.65, p=0.179) |  |
| **CD68** | CD68^low^ | 202 (50.0) |  |  |
|  | CD68^high^ | 202 (50.0) | 1.61 (1.19-2.18, p=0.002) |  |
| **CD44v6/CD68** | CD44v6^low^ / CD68^low^ | 100 (24.8) |  |  |
|  | CD44v6^low^ / CD68^high^ | 102 (25.2) | 1.61 (1.02-2.53, p=0.039) | 1.37 (0.87-2.15, p=0.178) |
|  | CD44v6^high^ / CD68^low^ | 102 (25.2) | 1.24 (0.77-1.99, p=0.382) | 1.11 (0.68-1.79, p=0.684) |
|  | CD44v6^high^ / CD68^high^ | 100 (24.8) | 2.02 (1.30-3.15, p=0.002) | 1.85 (1.18-2.89, p=0.007) |


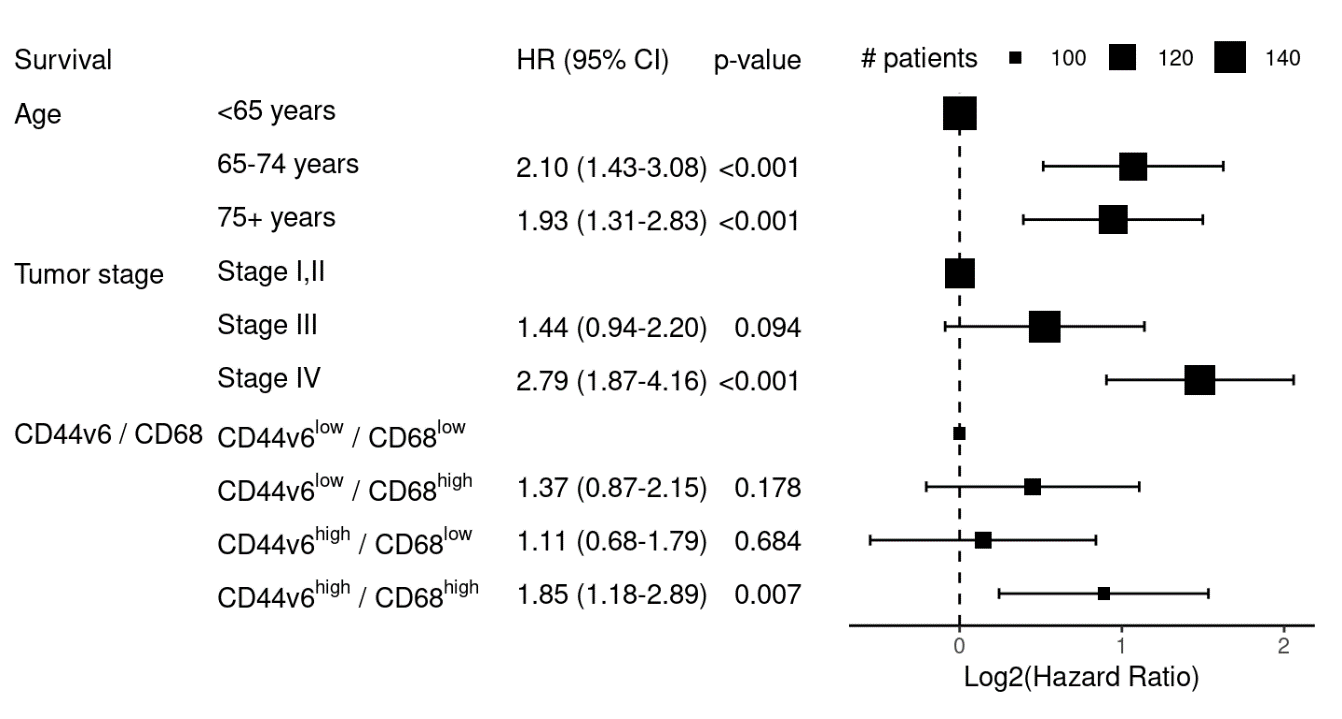


**Supplementary_Figure_4. Hazard ratio forest plots of multivariate analyses.** Each bar represents the hazard rate posed by one factor (age, tumor stage or combined CD44v6/CD68 levels) divided by the hazard rate caused by the lowest values of the factor. Each rectangle represents the overall ratio for all patients within a factor and the rectangle size is proportional to the number of patients considered. Horizontal bars represent the 95% confidence interval. The ratios are plotted on a logarithmic scale.

**Supplementary_Table_3: Cox-regression hazards ratio (HR) in univariate and multivariate analyses.**

| **Variable** |  | **Patients (percentage)** | **Univariate analysis** | **Multivariate analysis** |
| --- | --- | --- | --- | --- |
|  |  |  | **HR (95% CI, p-value)** | **HR (95% CI, p-value)** |
| **M0** | M0^low^ | 202 (50.0) | - | - |
|  | M0^high^ | 202 (50.0) | 2.07 (1.52-2.82, p<0.001) | - |
| **M1** | M1^low^ | 202 (50.0) | - | - |
|  | M1^high^ | 202 (50.0) | 0.90 (0.67-1.21, p=0.491) | - |
| **M2** | M2^low^ | 202 (50.0) | - | - |
|  | M2^high^ | 202 (50.0) | 0.80 (0.60-1.08, p=0.141) | - |
| **CD44v6/M0** | CD44v6^low^ / M0^low^ | 98 (24.3) | - | - |
|  | CD44v6^low^ / M0^high^ | 104 (25.7) | 1.63 (1.04-2.57, p=0.033) | 1.29 (0.81-2.05, p=0.279) |
|  | CD44v6^high^ / M0^low^ | 104 (25.7) | 0.96 (0.59-1.58, p=0.880) | 0.85 (0.51-1.40, p=0.527) |
|  | CD44v6^high^ / M0^high^ | 98 (24.3) | 2.53 (1.64-3.92, p<0.001) | 2.21 (1.42-3.46, p<0.001) |


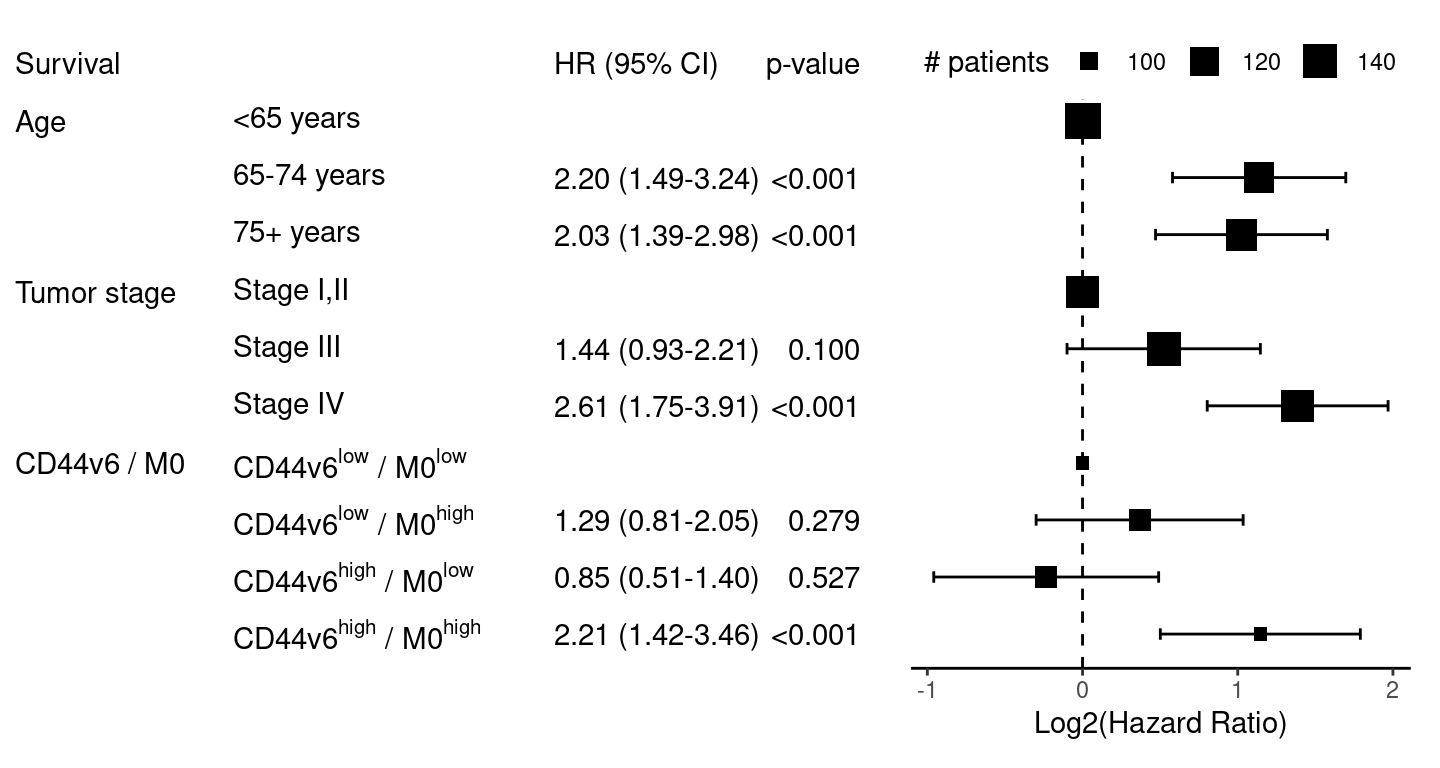


**Supplementary_Figure_5. Hazard ratio forest plots of multivariate analyses.** Each bar represents the hazard rate posed by one factor (age, tumor stage or combined CD44v6/M0 levels) divided by the hazard rate caused by the lowest values of the factor. Each rectangle represents the overall ratio for all patients within a factor and the rectangle size is proportional to the number of patients considered. Horizontal bars represent the 95% confidence interval. The ratios are plotted on a logarithmic scale.
